# Prevalence of violence towards men living with HIV/AIDS in rural communities of SouthWestern Uganda

**DOI:** 10.4314/ahs.v22i3.51

**Published:** 2022-09

**Authors:** Javilla Kamya Kakooza, Ritah Nampijja, Faith Kwagala, Flavia Nuwabasa, Owen Mpuuga, Gomer Isiagi, Godfrey Zari Rukundo

**Affiliations:** 1 Mbarara University of Science and Technology, Faculty of Medicine, P.O.Box1410, Mbarara city Uganda; 2 Department of Psychiatry, Mbarara University of Science and Technology, Faculty of Medicine, P.O.Box 1410, Mbarara city Uganda

**Keywords:** HIV positive men, violence, prevalence, rural communities, Uganda

## Abstract

**Background:**

Violence towards HIV positive men is one of the silent barriers to utilization of HIV care services. HIV positive men are potential victims of violence from other people including women, and violence may interfere with treatment outcomes. This study determined the prevalence of violence towards HIV positive men in rural communities of southwestern Uganda.

**Methods:**

A cross-sectional study was conducted among 307 HIV positive men at selected health centers using an interviewer administered questionnaire. Data were analyzed in SPSS version 23 using chi-square and multivariate regression at 95% level of significance and a precision of 0.05.

**Results:**

Of the 307 participants, 45.3% had experienced violence. Of these, 23.8% (n=73) had experienced kicking or slapping while 12.7% (39) reported sexual violence. Factors associated with violence were; using alcohol and drugs (aOR 0.26, 95% CI 0.09–0.76, p=0.014), knowledge of support structures (OR 2.25, 95% CI 1.33–3.78, p=0.002) and owning land for farming (aOR 0.26, 95% CI 0.10–0.70, p=0.011).

**Conclusion:**

The prevalence of violence at 45.3% is quite high especially since violence against men is rarely talked about. This should not be ignored there should be strategies to support this vulnerable group.

## Introduction

Violence has been defined by the World Health Organization as “the intentional use of physical force or power, threatened or actual, against oneself, another person, or against a group or community that either results in or has a high likelihood of resulting in injury, death, psychological harm, maldevelopment or deprivation 1. It is a phenomenon commonly studied among women 2–4. While a woman's violence is often seen as self-defense or as an expression of frustration and stress, men are often perceived as perpetrators 5, 6. Violence towards men is rarely talked about although some previous research in high-income countries shows that men equally suffer from violence and its consequences 7–10. Men often fear to disclose violence in their homes and communities because of ‘masculinity,’ tendency to be strong, resilient and being in control 11, 12. In sub-Saharan Africa, violence towards men is highest in war-torn countries with a prevalence of up to 64.5% 13. Violence is more pronounced among HIV positive men and the HIV positive status is one of the risk factors 14. While the intersection of HIV/AIDS and violence has gained momentum over the years, much focus is on women and children with little attention to HIV positive men 14, 15. According to previous studies, HIV can also be a risk factor for violence since disclosure of a positive status can put the individual at risk of violence by their partners, family or community members. However, most previous studies have focused on violence towards women 16, 17. Violence towards the HIV positive men can be perpetrated from the family and the community at large because of different reasons including their HIV positive status 14, 18, 19.

People living with HIV and experiencing violence in rural communities may be at increased risk for adverse clinical outcomes associated with HIV diagnosis and inadequate management resources 14, 20. Violence perpetrated in rural communities may be more chronic and may have worse physical, psychological and social health outcomes due to the disparities in access and availability of services 21–24 . When violence towards HIV positive men if not attended to it may be associated with depression, non-adherence to ARVs, lost follow-up, family neglect and suicide 13, 25. With the high HIV prevalence in western Uganda, the aim of the present study was to investigate the prevalence, forms and factors associated with violence towards HIV positive men attending HIV care in health facilities in Bushenyi district in southwestern Uganda. In this study we adapted he world Health Organisation definition of violence. We defined violence against men as “the intentional use of physical force or power, threatened or actual, against men, which either resulted in or had a high likelihood of resulting in injury, death, psychological harm or deprivation” 1. We chose to conduct the study in HIV clinics because we needed to be sure that the HIV diagnosis was confirmed. Given the stigma of HIV, it would have been difficult to identify the men living with HIV.

## Methods

### Study design

This was an analytical cross sectional study conducted in April 2021.

### Study setting

The structure of the Ugandan health care system includes the community level services by the village health teams; health centers (HC II, at parish level; HC III, at sub-county level; and HC IV, at county level), district level (general) hospitals, regional referral hospitals, and national referral hospitals 26. The HC II provides only ambulatory services except in strategic locations such as poor access to HCIII or HC IV where as interim strategy maternity services are being provided. The HC III offers continuous basic preventive, promotive and curative care and provides support supervision of the community and HCII facilities. In addition to services provided by HC II, there are provisions for laboratory services for diagnosis maternity care and HIV care. The HC IV offers services similar to those at HC III. However, in addition, they have an operating theatre and they supervise the HC III. The HC IV has medical doctors and other high cadre health professionals 26. All HIV services at the various levels of care are offered at no cost in public health facilities.

The study was conducted in Igara county, Bushenyi district, southwestern Uganda at four health centers: Kyabugimbi HC IV, Ruhumuro HC III, Bitooma HC III and Kakanju HC III. We recruited participants from various health facilities so as to raise a study. There was no intention to compare the samples from the different facilities. Igara County is 463 kilometers from Kampala city along Mbarara-Kasese highway in southwestern Uganda. The main economic activity is mainly small scale agriculture and retail to commercial businesses. The area is occupied mainly by the Banyakore-Bakiga tribe whose native language is Runyakole-Rukiga. Kyabugimbi HC IV is located 6.1km off the main road and is found between Kyabugimbi and Bugalama trading centers. Kakanju HC III is 16 km away while Ruhumuro is 26 km North West away from Kyabugimbi trading center and Bitooma HC III is 22 km North East of Kyabugimbi.

### Study population

The study was conducted among 307 adult HIV positive men attending HIV care at the four health centers (Kyabugimbi, Ruhumoro, Bitooma and Kakanju). The sample was calculated basing on the prevalence of 22.4% in a previous 27. According to the Kish and Leslie formula, the minimum required sample was 303 HIV positive men.

### Data collection procedure

The participants were recruited from the four facilities in Igara County, Bushenyi district. The participants were consecutively recruited until the required number was obtained. The clinics were on different days at the different health facilities. We used an interviewer administered questionnaire to collect data on sociodemographic characteristics (age, marital status, Ownership of land and other items, occupation and the level of education), violence and substance use. The questions on violence were adapted from the Abuse Assessment Screen^28^. The questionnaire was administered by the research assistants. The research assistants (Nurse and social scientist) were trained by the senior author on how to administer the questionnaire and have previously participated in community research projects. The questionnaire has not been formally validated for Uganda but it was translated into the local language (Runyankore-Rukiga) and pretested among 20 HIV positive men receiving HIV care from Mbarara Regional Referral Hospital and found to be appropriate. Research assistants were supervised by the senior author. With the help of the HIV clinic in-charges, HIV positive men who had come for care were identified. Using consecutive sampling method, the participants who fulfilled the inclusion criteria were requested by the research assistants to participate in the study. The research assistants introduced the study to potential participants and asked them for consent. The participants were informed that participation was voluntary and that they were free to stop their participation at any point without any negative consequences. Those who accepted to participate signed consent forms and thereafter completed the questionnaire which took between 20–30 minutes. The participants who were not able to read and write used their thumb prints to confirm their consent to participate.

### Variables

The dependent variable was violence due the HIV positive status among HIV positive men attending care in health facilities in Bushenyi district southwestern Uganda. We assessed the forms, associated factors and perceptions about violence due to HIV positive status.

The independent variables were; Age, marital status, education level, level of income, occupation, duration of HIV diagnosis, substance use, perceptions of HIV positive men towards violence, availability of support structures (Family divisions in police), and access to the support structures.

### Ethical considerations

The study protocol was reviewed and approved by the Mbarara University Research Ethics Committee No.06/12-20. Administrative clearance to conduct the study was obtained from the Bushenyi District Health Officer. Details of the power of choice to participate were explained to the participants. The respondents were informed that they were free to refuse to respond to questions that they did not feel comfortable answering. Informed written consent was obtained from all the participants before enrolling for the study. For purposes of confidentiality, the respondent's names were not recorded on the questionnaires, but codes were allocated to each participant.

### Data management and analysis

While in the field, we ensured completeness of the questionnaires before the respondents left the interview rooms. The data were later entered into SPSS version 23 and cleaned before analysis. Categorical variables were analyzed using chi-square and multivariate regression at 95% confidence interval. At multivariate regression to determine variables that were independently associated with the outcome variable, the potential confounders were included (age, marital status, level of education, employment, knowledge about available support services and access to the support services). The model fitness was tested using Hosmer-Lemeshow test at p>0.05.

## Results

### Participant characteristics

Of the 307 participants in the study, 35.5% (109/307) were in the age group 36–45 years, 59.6% (183/307) had completed only primary education and 32.9% (101/307) were unemployed (Table 1).

**Table 1:** Participant characteristics and prevalence of violence among HIV positive men attending selected health centers in Bushenyi district (N= 307), April, 2021

Characteristic variable	Description	Frequency, N=307, n(%)	Experienced violence n(%) 139 (45.3%)	Didn't experience violence n (%) 168 (54.7%)
Age	18–24	28(9.1)	17(12.2)	11(6.5)
	25–35	80(26.1)	34(24.5)	46(27.4)
	36–45	109(35.5)	48(34.5)	61(36.3)
	46–60	70(22.8)	35(25.2)	35(20.8)
	>60	20(6.5)	5(3.6)	15(8.9)
Education level	No formal education	44(14.3)	18(12.9)	26(15.5)
	Primary education	183(59.6)	84(60.4)	99(58.9)
	Secondary	67 (21.8)	32(23.0)	35(20.8)
	Tertiary/University	13(4.2)	5(3.6)	8(4.8)
Marital status	Never married	43(14.0)	23(16.5)	20(11.9)
	Married monogamous	140(45.6)	61(43.9)	79(47.0)
	Married polygamous.	48(15.6)	27(19.4)	21(12.5)
	Widowed or divorced	76(24.8)	28(20.1)	48(28.6)
Ownership of land and other items	Doesn't own land	56(18.2)	35(25.2)	21(12.5)
	Owns land for farming	195(63.5)	82(59.0)	113(67.3)
	Owns land and motorcycles or cars	56(18.2)	22(15.8)	34(20.2)
Occupation	Unemployed	101(32.9)	44(31.7)	57(33.9)
	Business	55(17.9)	24(17.3)	31(18.5)
	Formal employment	17(5.5)	8(5.8)	9(5.4)
	Informal employment	134(43.6)	63(45.3)	71(42.3)

### Prevalence of violence towards HIV positive men attending selected health centers in Bushenyi district

The prevalence of violence towards HIV positive men was 45.3% (n=139/307, 95% CI 39.4–51.1) and 43.9% (61/139) were in monogamous relationships (Table 1). In our study sample, 23.8% (n=73) experienced physical violence (kicking or slapping) and 39 (12.7%) men reported sexual violence. Of these HIV positive men, 51 (69.9%) reported being afraid of their perpetrators. The perpetrators of the slapping or kicking were: fellow men (41.1%, n=30), strangers (30.1%, n=22), current wives (13.7%, n=10), former wives (8.2%, n=6) and girlfriends (6.9%, n=5). Thirteen individuals (17.8%) reported being hit on the head while 29 (39.7%) reported being hit on the abdomen or chest.

### Predictors of violence towards HIV positive men attending selected health centers in Bushenyi district

From the multivariate analysis, use of alcohol and smoking as well as ownership of land for farming were independently associated with increased risk for violence among HIV positive men (Table 2).

**Table 2:** Predictors of violence among HIV positive men attending selected health centers in Bushenyi district (N= 307), April, 2021

Variable characteristic	Description	Unadjusted Prevalence ratio	95 % CI	p-value	Adjusted Prevalence ratio	95 % CI	p-value
Age (years)	18–24	1					
	25–35	2.43	1.07–5.49	0.03	2.01	0.83–4.86	0.121
	36–45	1.70	0.76–3.77	0.19	1.57	0.74–3.32	0.237
	46–60	1.76	0.80–3.87	0.16	1.59	0.76–3.32	0.217
	< 60	2.00	0.90–4.43	0.09	1.91	0.93–3.91	0.076
Level of education	No formal education	1					
	Primary	1.064	0.49–2.31	0.88	1.49	0.47–4.71	0.495
	Secondary	1.19	0.59–2.42	0.62	1.37	0.45–4.16	0.578
	Tertiary/ university	1.24	0.60–2.58	0.56	1.27	0.44–3.69	0.662
Occupation	Unemployed	1					
	Business	0.93	0.70–1.23	0.60	0.93	0.67–1.29	0.662
	Formal employment	0.93	0.65–1.32	0.68	1.01	0.73–1.42	0.938
	Informal employment	1.00	0.59–1.71	0.10	1.12	0.49–2.57	0.788
Marital status	Never married	1					
	Married-Monogamous	1.45	0.97–2.18	0.07	1.13	0.61–2.10	0.703
	Married-polygamous	1.18	0.83–1.68	0.35	0.99	0.67–1.45	0.937
	Separated/divorced	1.53	1.04–2.25	0.03	1.40	0.93–2.13	0.111
Level of income (Ownership of land and other items)	Doesn't own land	1					
	**Owns land for** **farming**	**1.59**	**1.08–2.34**	**0.02**	**1.79**	**1.20–2.66**	**0.004**
	Owns land and motorcycles or cars	1.07	0.74–1.54	0.72	1.31	0.94–1.83	0.111
Use of substance (alcohol and smoking)	Doesn't use any	1					
	Use one (either alcohol or smoking)	1.09	0.58–2.03	0.79	1.32	0.71–2.47	0.380
	**Use of both** **alcohol and** **smoking**	**1.78**	**0.96–3.31**	**0.07**	**1.95**	**1.04–3.66**	**0.039**
Knowledge of the support structures	No	1					
	Yes	0.69	0.53–0.91	0.01	2.45	0.35–17.05	0.364
Access to support structures	No	1					
	Yes	0.64	0.49–0.85	0.00	0.26	0.04–1.82	0.174

## Discussion

The aim of the present study was to investigate the prevalence and factors associated with violence towards HIV positive men attending in health facilities of Bushenyi district in southwestern Uganda. We found that almost half of the HIV positive men had experienced violence within the previous 3 months. The factors associated with violence towards HIV positive men included ownership of land for farming and substance use.

The prevalence of 45.3% is quite high yet in practice it is usually undetected. This is higher than the prevalence of 39% reported by Bryan and colleagues among HIV positive men in a rural setting in Appalachia USA 29. The difference could be due to the disparity in study tools and settings. In addition, the study by Bryan and colleagues focused on intimate partner violence but ours also considered violence from different individuals including the spouses of the victims. The prevalence of violence in our study was also higher than that of Wang and colleagues who also focused on only intimate violence among men who have sex with men 30. Apart from intimate partner violence, HIV positive men experience other forms of violence that need to be studied. As expected, the prevalence in our study is lower than that among women 31 in which men are the most common perpetrators 31.

Several factors are associated with violence in HIV positive individuals 32–34. The factors associated with violence are common in the community and seem to be the very factors associated with violence in the general population. Generally, men are considered to be perpetrators of violence and some of the associated factors are socio-economic. According to our findings, having a good Ownership of land and other items was protective against violence. This could be to the fact that individuals with a higher socioeconomic status are respected by the members of the community and therefore not violated even when they are HIV positive.

At multivariate analysis, consumption of alcohol and other drugs was associated with increased risk of violence. This is similar to previous studies that have associated substance use with perpetration of violence 35, 36. It is possible when they are under the influence of substances they become victims of violence. It is also possible that the individuals who have suffered violence may resort to substance use as a coping mechanism. Thereis need for further exploration of this relationship.

Owning land for farming was found to be to be associated with violence among HIV positive men. These being rural communities, the desire to own land may lead to land wrangles between family and community members since it a treasured and prestigious possession. Farming is also associated with having many things to do and with less time for redundancy. Having land for farming protects against food insecurity and poverty which are risk factors for violence in homes 37–39.

At bivariate analysis, it was surprising to find that having knowledge of the available support structures predicted violence among HIV positive. We did not ask how many times they have accessed the support structures. It is possible that these victims of violence have already accessed the support structures and therefore knowledge about them. Victims of violence often have vulnerability factors that put them at risk of experiencing violence several times. In a previous Ugandan study, men were reported as having less enthusiasm for seeking care 40. However, the association did not remain significant after controlling for the confounding factors.

## Implications of the findings

Although men have been reported as perpetrators of violence among women, they are also victims. Unfortunately, their vulnerability is usually not assessed in routine care. There is a need to regularly assess men for the risk violence^41^. For example, some questions on violence may be incorporated in the routine assessment form so that clinicians are reminded to ask about violence among men seeking care. The regular screening for violence against men may help identify those at risk for poor health outcomes so as to mitigate the negative effects. If violence against men is not assessed and managed it can lead to many complications such as poor treatment adherence and further spread of HIV 42. In addition, there is need for more research on violence among HIV positive many in sub-Saharan Africa which carries the biggest burden of HIV and violence 43.

## Limitations of the study

We did not assess the extent to which violence among the HIV+ men differs from that in HIV-negative men since all our study participants were HIV positive. The direction of the association between substance use and violence was not specified to confirm if the substance use is a risk factor or a consequence of violence. Being a cross-sectional study, we are unable to confirm this. In addition, we did not assess for sexual orientation in this study. It is possible that some of the men could be having sex with other men, a group that has more risk of sexual abuse by other men. Since data were collected from multiple health centers, at analysis, there could be design effect although all the health facilities are in the same locality. The design effect could have reduced the precision of our sample estimate.

## Conclusion

There is a high prevalence of violence towards the HIV positive men southwestern Uganda. Policy makers and other relevant stakeholders need to put strategies in place to support this vulnerable group.

## Data Availability

All the data needed for this manuscript has been included. In case there is a need for clarifications, the corresponding author can be contacted.
